# Evaluation of the morphological features of the uterine tubes during postnatal development in West African Dwarf goats (*Capra hircus*)

**Published:** 2017-03-15

**Authors:** Clifford Nwabugwu Abiaezute, Innocent Chima Nwaogu, Udensi Maduabuchi Igwebuike

**Affiliations:** *Department of Veterinary Anatomy, Faculty of Veterinary Medicine, University of Nigeria, Nsukka, Nigeria.*

**Keywords:** Mucosal fold, Postnatal development, Puberty, Uterine tube, West African Dwarf goat

## Abstract

The objective of this study was to highlight the postnatal development of the uterine tubes of the West African Dwarf goat from birth to 28 weeks of age by gross examination and light microscopy. There was a caudal migration of the paired uterine tubes from behind the paired kidneys at birth to the pelvic inlet at week 8 of age. Each uterine tube exhibited three segments namely; infundibulum, ampulla and isthmus. A marked flexure, the utero-tubal junction, was the point at which the uterine tubes joined the uterine horns. The length and absolute weight of the uterine tubes increased from 4.95 ± 0.28 cm and 0.02 ± 0.01 g at birth to 14.98 ± 2.79 cm and 0.22 ± 0.03 g at week 28 of age, respectively. The mucosa of the infundibulum and the ampulla showed long, branched and anastomosing primary, secondary and tertiary mucosal folds which decreased in height towards the isthmus. The mucosal folds within the isthmus were short and lacked the anastomosing pattern. The epithelia of all three segments were pseudostratified columnar. Numerous secretory blebs and extruded nuclei became apparent from week 16 of age. The thickness of the tunica muscularis varied with the segments.

## Introduction

Mammalian uterine tubes are paired, highly convoluted tubes of paramesonephric duct origin that connect the paired ovaries to the rest of the reproductive tract. The uterine tubes are suspended within the mesosalpinx of the broad ligament and constitute an important part of the reproductive tract in female animals because they serve as a duct for transport of spermatozoa, oocytes and fertilized ova. Furthermore, uterine tubes provide the suitable microenvironment for spermatozoa storage, capacitation, fertilization and early cleavage-stage embryonic development.^1^^,^^2^ The uterine tubes are connected to the uterus and both structures offer a great diversity of morphological and functional features in different species of animals with respect to their reproductive and evolutionary adaptation.^3^ It is common knowledge that mammalian uterine tubes consist of three anatomical regions namely, infundibulum, ampulla and isthmus, each of which is associated with distinct physiological functions.

 A large body of knowledge is available about the morphology of the ovaries and uterine bodies of domestic animals,^4^^-^^9^ but there is paucity of information about the morphology of the goat uterine tubes. Moreover, most descriptions of goat anatomy and physiology are based on assumptions of similarities between the goat and the sheep.^10^^,^^11^

 Therefore, the present study seeks the morphological changes that may occur in the structure of the uterine tubes during postnatal development in West African Dwarf (WAD) goats; a miniature goat breed of West African origin.

## Materials and Methods

 Forty five female WAD goats of known ages were used in this study. The female goats were obtained from traditional WAD goat breeders in Nsukka Local Government Area of Enugu state, Nigeria. The WAD goats were purposively assigned to nine groups of five goats each including day old, 2, 4, 8, 12, 16, 20, 24 and 28 weeks of age. Each goat was weighed with a sensitive weighing balance (Model BR9010; Guangdong, China) and euthanized by intravenous injection of 70 mg kg^-1^ sodium pentobarbitone (Kyron Laboratories Ltd., Johannesburg, South Africa) at body weight. Following death, the uterine tubes were dissected free from the rest of the reproductive tract and trimmed of extraneous tissues. The length and weight of each uterine tube were determined.

Segments of the infundibulum, ampulla and isthmus of each uterine tube were cut and fixed by immersion in Bouin’s fluid for 24 hr. Sections of the segments from each group were dehydrated in increasing concentrations of ethanol, cleared in xylene and embedded in paraffin wax. Five µm thick sections were obtained using a. rotary microtome (Model 1512; Leitz^®^, Wetzlar, Germany) and mounted on clean glass slides. The sections were stained with hematoxylin and eosin and studied under the light microscope. The heights of six non-anastomosing mucosal folds and thickness of the tunica muscularis of each segment for each group were measured using the ocular micrometer gauge after calibrating with the stage micrometer gauge at 100× magnification. Images were captured using Moticam Camera 1000 (Motic China group Ltd., Xiemen, China).

The means and standard errors (Mean ± SE) of the obtained data were calculated. The data were analysed by one way analysis of variance (ANOVA) and Duncan new multiple range test using SPSS (version 15.0; SPSS Inc., Chicago, USA). Significance was accepted at probability level of p < 0.05.

## Results

 Gross anatomical features. The uterine tubes appeared as paired tightly convoluted narrow tubes that coursed from the paired ovaries to the tip of the paired uterine horn. At birth, the paired uterine tubes and the paired ovaries of WAD goats were located caudal to the paired kidneys in the sub-lumber region of the abdominal cavity. The uterine tubes were located midway between the paired kidneys and the pelvic inlet at week 4 of age but were completely within the pelvic inlet at week 8 of age. From birth until week 4 of age, the uterine tubes appeared creamy in colour, but at week 8 of age, the creamy colour of the uterine tubes exhibited some pinkish patches and by week 16, the uterine tube was uniformly pink in colour. Each uterine tube exhibited three segments namely; infundibulum, ampulla and isthmus. The infundibulum, which was the closest segment to the ovary was funnel-shaped and possessed irregular finger-like projections, the fimbriae that wrapped around the ovary. The middle segment, the ampulla was the longest segment of the uterine tube and coursed caudally from the infundibulum to the isthmus. The isthmus was a short caudal segment of the uterine tube that joined the uterine horn at a marked flexure, the utero-tubal junction. The length of the left and right uterine tubes showed statistically significant increases (p < 0.05) from birth to week 28, respectively (Table 1). The weight of the left and right uterine tubes showed statistically significant increases (p < 0.05) from birth to week 28. The relative organ weight of the uterine tubes at the different ages showed no significant differences. There were no significant differences (p > 0.05) between the length and weight of the left uterine tubes and those of the right ones within studied groups.

**Fig. 1:**
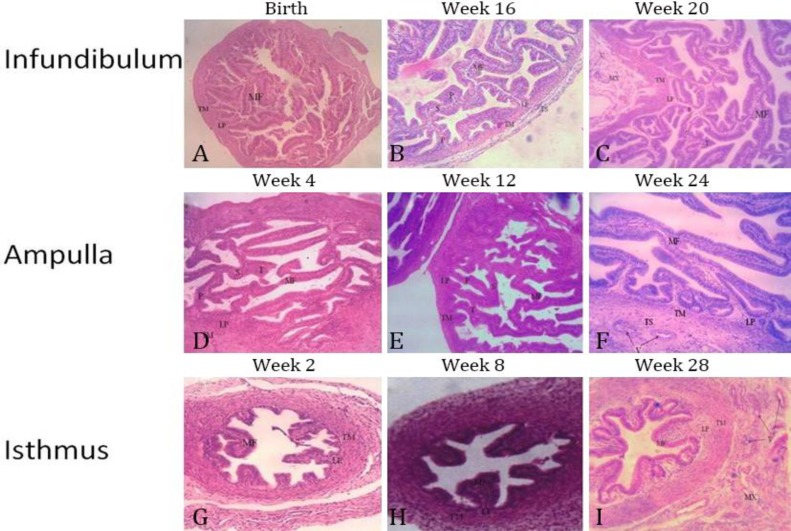
Sections of infundibulum, ampulla and isthmus at different ages of WAD goats showing the anastomosing pattern of the uterine mucosal folds (MF) extending from the propria-submucosa (LP). Note the primary (P), secondary (S) and tertiary (T) mucosal folds. Also, note the tunica muscularis (TM), tunica serosa (TS) and blood vessels (V) within the mesosalpinx (MX), (H & E, 100×).

**Fig. 2:**
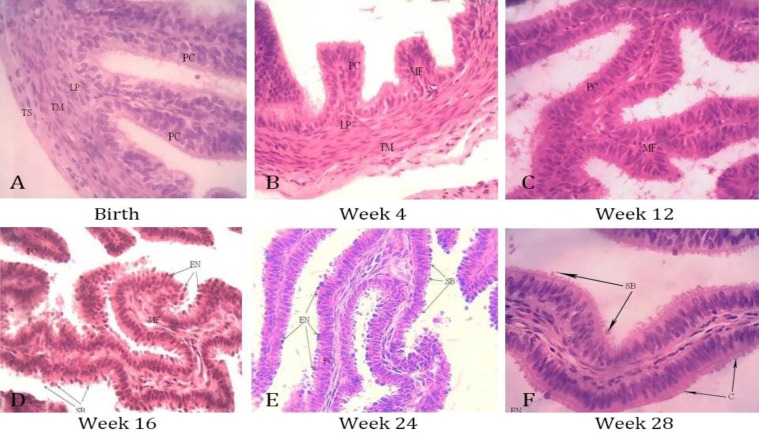
Photomicrograph of uterine tube mucosal folds (MF) at different ages showing the pseudostratified columnar epithelium (PC). Note the numerous secretory blebs (SB) and extruded nuclei (EN). Also, note the propria-submucosa (LP), tunica muscularis (TM) and the tunica serosa (TS) (H & E, 400×).

Histology. In all groups, the tunica mucosa of infundibulum and ampulla exhibited numerous long branched mucosal folds extending into the lumen of the uterine tube. These primary mucosal folds gave rise to secondary mucosal folds which occasionally extended as tertiary mucosal folds. The secondary and tertiary mucosal folds anastomosed with adjacent similar folds and occasionally opposite mucosal folds creating a complex basket-like arrangement that almost occluded the lumen of the proximal and middle segments of the uterine tube (Fig. 1 A to F). The mucosal folds in the isthmus were not as tall as those found in the infundibulum and ampulla (Table 2) and they also lacked the anastomosing pattern (Fig. 1 G to I). The epithelial lining of the tunica mucosa of all segments of the uterine tube was tall pseudo-stratified columnar epithelium (Fig. 2). From birth until week 12 of age, there was no clear evidence of ciliated or secretory cells in the epithelium. However, by week 16, the secretory and ciliated cells were apparent with numerous extruded nuclei and secretory blebs appearing within the uterine tube lumen (Fig. 2). These secretory and ciliary activities were observed intermittently in subsequent preceding groups.

**Table 1:** Mean length, mean weight and relative weight of the uterine tubes of WAD goats during postnatal growth.

	**Birth**	**Week 2**	**Week 4**	**Week 8**	**Week 12**	**Week 16**	**Week 20**	**Week 24**	**Week 28**
***Mean length (cm)***									
**Left**	4.95 ± 0.28^a^	5.48 ± 0.24^a^	7.47 ± 0.54^ab^	9.74 ± 1.38^bc^	10.62 ± 0.76^bcd^	11.20 ± 0.61^bcd^	12.30 ± 1.71^cd^	13.70 ± 1.93^cd^	14.98 ± 2.79^d^
**Right**	4.96 ± 0.26^a^	5.50 ± 0.26^a^	7.62 ± 0.61^ab^	9.79 ± 1.13^bc^	10.90 ± 0.70^bcd^	11.20 ± 0.71^bcd^	12.60 ± 1.32^cde^	14.00 ± 1.66^de^	15.04 ± 2.50^e^
***Mean weight (g)***									
**Left**	0.02 ± 0.01^a^	0.03 ± 0.01^ab^	0.04 ± 0.00^abc^	0.07 ± 0.01^bc^	0.08 ± 0.01^cd^	0.12 ± 0.01^de^	0.13 ± 0.02^e^	0.20 ± 0.02^f^	0.22 ± 0.03^f^
**Right**	0.02 ± 0.01^a^	0.03 ± 0.01^a^	0.04 ± 0.01^ab^	0.07 ± 0.01^bc^	0.09 ± 0.01^c^	0.11 ± 0.01^cd^	0.13 ± 0.02^d^	0.20 ± 0.02^e^	0.22 ± 0.02^e^
***Relative weight (%)***									
**Left**	0.01 ± 0.00^a^	0.01 ± 0.00^a^	0.01 ± 0.00^a^	0.02 ± 0.00^a^	0.02 ± 0.00^a^	0.02 ± 0.00^a^	0.01 ± 0.00^a^	0.02 ± 0.00^a^	0.02 ± 0.00^a^
**Right**	0.01 ± 0.00^a^	0.01 ± 0.00^a^	0.01 ± 0.00^a^	0.02 ± 0.00^a^	0.02 ± 0.00^a^	0.02 ± 0.00^a^	0.01 ± 0.00^a^	0.02 ± 0.00^a^	0.02 ± 0.00^a^

Different superscripts in a row indicate significant difference (*p *≤ 0.05).

The results of this study also showed that the connective tissue of the lamina propria blends with the submucosa in the walls of the three segments to constitute the propria-submucosa which was extended to form the core of the mucosal folds (Fig. 2). In all groups, the tunica muscularis was thinly developed in the infundibulum, moderately developed in the ampulla and the thickest in the isthmus (Table 2). The thickness of the tunica muscularis increased with age within each segment. The tunica serosa as a loose connective tissue was lined by a simple squamous epithelium on its external surface. The uterine tube was invested in a thin transparent fold of peritoneum, the mesosalpinx with numerous blood vessels (Fig. 1).

**Table 2:** Histomorphometric measurements of the uterine tube mucosal folds and tunica muscularis in different segments during postnatal development of WAD goats.

	**Birth**	**Week 2**	**Week 4**	**Week 8**	**Week 12**	**Week 16**	**Week 20**	**Week 24**	**Week 28**
***Mean mucosal fold height (µm)***									
**Infundibulum**	61.30 ± 6.30^a^	67.10 ± 4.20^ a^	78.60 ± 6.50^b^	87.40 ± 5.70^bc^	95.20 ± 6.90^c^	137.70 ± 10.40^d^	148.20 ± 14.60^d^	156.80 ± 13.20^d^	162.50 ± 16.70^d^
**Ampulla**	48.90 ± 5.70^a^	56.00 ± 5.10^ab^	67.20 ± 7.40^bc^	75.80 ± 6.80^ c^	89.30 ± 6.30^d^	119.50 ± 9.60^e^	130.10 ± 7.5^ef^	138.20 ± 8.80^f^	146.40 ± 11.10^ f^
**Isthmus**	8.20 ± 1.10^a^	9.50 ± 1.30^ab^	10.30 ± 1.70^ab^	11.80 ± 1.60^bc^	12.90 ± 2.10^bcd^	15.10 ± 2.20^cde^	16.70 ± 1.80^de^	18.10 ± 2.80^e^	19.00 ± 2.60^e^
***Tunica muscularis thickness (µm)***									
**Infundibulum**	0.80 ± 0.10^a^	0.80 ± 0.10^a^	1.00 ± 0.20^ab^	1.30 ± 0.30^bc^	1.80 ± 0.40^c^	3.00 ± 0. 30^d^	3.70 ± 0.40^de^	4.30 ± 0.50^e^	4.90 ± 0.40^e^
**Ampulla**	1.40 ± 0.20^a^	1.60 ± 0.20^a^	2.50 ± 0.20^b^	3.20 ± 0.20^c^	4.00 ± 0.40^d^	5.80 ± 0.40^e^	6.40 ± 0.40^ef^	7.00 ± 0.40^ f^	7.80 ± 0.60^f^
**Isthmus**	2. 80 ± 0.40^a^	3.50 ± 0.30^a^	4.60 ± 0.30^b^	6.30 ± 0.50^c^	8.40 ± 0.60^d^	11.20 ± 1.10^e^	13.10 ± 0.70^f^	14.40 ± 0.70^g^	15.60 ± 1.10^h^

Different superscripts in a row indicate significant difference (*p *≤ 0.05).

## Discussion

The present study illustrates morphological evidence for caudal migration of the uterine tubes and ovaries of WAD goats from their initial position behind the kidneys at birth to the pelvic inlet by week 8 of age. This caudal migration of the uterine tubes may be relative owing to increase in body length as the goat grows, or may result from contraction of the gubernaculum and its derivatives. Caudal migration of foetal ovaries and attached uterine tubes from their initial sites of formation in the thoraco-lumber region has been reported by previous authors.^12^^,^^13^ Moreover, it has been demonstrated that just before parturition, foetal ovaries of Kano brown goats are located caudal to the kidneys.^8^ This supports the idea that the final caudal migration of the ovaries and the attached uterine tubes into the pelvic inlet in goats occurs postnatally as shown in the present study.

 The gross morphology of the uterine tube of the WAD goat is similar to that of other ruminants; in that it consists of the fimbriated funnel-shaped infundibulum, ampulla and isthmus.^14^ There was a gradual statistically significant growth in length and weight of the uterine tubes with age. However, the relative contribution of the uterine tubes to the overall weight of the animal did not vary significantly as the goats progressed in age. This suggests that the uterine tube growth was commensurate with the growth of the animal.

Histologically, the wall of the uterine tubes of female WAD goats exhibited the typical four layered tissues of uterine tubes of ruminants.^15^^-^^17^ The result of this study showed decreasing mucosal folds height from the infundibulum to the isthmus. This findings probably confirm the role of infundibulum in capturing and channelling of the oocyte from the ovary to the ampulla.^2^^,^^3^ The tall, branched and anastomosing mucosal folds of the infundibulum and ampulla of WAD goat differ from the reported pattern of regular tall, branched and highly folded mucosal folds of ruminants.^2,15,18^ These tall and anastomosing mucosal folds in the present study invariably resulted in increased mucosal surface area and may also serve to slow down the movement of the oocyte or zygote towards the isthmus. It has been reported that newly ovulated oocytes are transported rather slowly within the uterine tubes of ewes (72 hr) and cows (90 hr).^19^ Such delay within the proximal segments of the uterine tube may permit further maturation of the oocyte or further development of the zygote to attain the morula stage prior to its arrival at the uterus. Premature entry of the morula into the uterus has been shown to result in embryo loss.^19^^-^^21^ However, occurrence of short mucosal folds in the wall of the isthmus suggests that delay in the transport of the embryo towards the uterus is not needful in this distal segment of the uterine tube.

The epithelial of all segments of the uterine tube were pseudostratified columnar. Ciliary and secretory activities were not observed in the epithelium of all segments of the uterine tube from birth until week 16 of age when extruded nuclei and secretory blebs were observed. This suggests that the WAD goat at week 16 may be in the follicular phase of oestrus. Secretory activities are known to increase in uterine tubes during the follicular stage of oestrus, under the influence of oestrogen.^2^^,^^19^^,^^22^ The presence of secretions in the uterine tubes of WAD goats from week 16 of age may be evidence of early attainment of puberty in WAD goats. Although, it has been reported that first oestrus in WAD goats occurs at 5 to 7 months of age,^23^ more recent reports have shown that goat may attain puberty as early as 3-4 months of age.^24^^,^^25^

Our study demonstrated that tunica muscularis within the walls of all three segments of the uterine tube in all groups is similar to those in ruminants.^2^^,^^17^^,^^22^ The varying thickness of the smooth muscles in the three segments suggests that the development of the smooth muscles of the different segments of the uterine tube may be related to the function of the segment. The thin to moderate layers observed in the infundibulum and ampulla may be related to the mild contractions within these segments described as a gentle ‘rocking’ or ‘to and fro’ type of contraction.^19^ However, the contractions of the thick tunica muscularis of the isthmus may result in stenosis of the flexure at the utero-tubal junction leading to restriction and regulation of the upward passage of sperm cells or downward transport of oocyte and fertilized zygote.^26^^,^^27^ Furthermore, the thick tunica muscularis probably serves to provide both peristaltic and anti-peristaltic waves in the isthmus. It has been reported that both peristaltic and antiperistaltic waves occur in the walls of the isthmus.^2^^,^^19^ Whereas, the antiperistaltic contraction of the smooth muscles will aid the sperm cells ascent towards the ampulla against ciliary movement.^1^^,^^2^^,^^28^^,^^29^ Peristaltic contractions of these smooth muscles together with movement of the cilia will help in propelling the early embryo towards the uterus.^2^^,^^30^

In conclusion, the present study has demonstrated that the final caudal migration of the uterine tubes occurs postnatally in WAD goats. The study also indicates early attainment of puberty in WAD goats than previously reported. Moreover, the wall of the different segments of the uterine tube exhibited unique morphological adaptations for their different functions.
